# Intradermal Administration of the Type II Heat-Labile Enterotoxins LT-IIb and LT-IIc of Enterotoxigenic *Escherichia coli* Enhances Humoral and CD8^+^ T Cell Immunity to a Co-Administered Antigen

**DOI:** 10.1371/journal.pone.0113978

**Published:** 2014-12-23

**Authors:** John C. Hu, Camila Mathias-Santos, Christopher J. Greene, Natalie D. King-Lyons, Juliana F. Rodrigues, George Hajishengallis, Luís C. S. Ferreira, Terry D. Connell

**Affiliations:** 1 Department of Microbiology & Immunology and The Witebsky Center for Microbial Pathogenesis and Immunology, The University at Buffalo, Buffalo, New York, United States of America; 2 Department of Microbiology, Institute of Biomedical Sciences, University of São Paulo, São Paulo, Brazil; 3 University of Pennsylvania School of Dental Medicine, Department of Microbiology, Philadelphia, Pennsylvania, United States of America; University Dresden, Germany

## Abstract

Vaccinations are extremely effective at combating infectious diseases. Many conserved antigen (Ag) targets, however, are poorly immunogenic. Protein subunit vaccines frequently elicit only humoral immune responses and fail to confer protection against serious intracellular pathogens. These barriers to vaccine development are often overcome by the use of appropriate adjuvants. Heat-labile enterotoxins (HLT) produced by enterotoxigenic strains of *Escherichia coli* are potent adjuvants when administered by mucosal or systemic routes. The efficacy of the type II HLT, however, has not been well-defined when administered by the intradermal (ID) route. Using a murine ID immunization model, the adjuvant properties of LT-IIb and LT-IIc, two type II HLTs, were compared with those of LT-I, a prototypical type I HLT. While all three HLT adjuvants enhanced Ag-specific humoral responses to similar levels, LT-IIb and LT-IIc, in contrast to LT-I, induced a more vigorous Ag-specific CD8^+^ T cell response and proffered faster clearance of *Listeria monocytogenes* in a challenge model. Additionally, LT-IIb and LT-IIc induced distinct differences in the profiles of the Ag-specific CD8^+^ T cell responses. While LT-IIc stimulated a robust and rapid primary CD8^+^ T cell response, LT-IIb exhibited slower CD8^+^ T cell expansion and contraction kinetics with the formation of higher percentages of effector memory cells. In comparison to LT-I and LT-IIc, LT-IIb evoked better long-term protection after immunization. Furthermore, LT-IIb and LT-IIc enhanced the total number of dendritic cells (DC) in the draining lymph node (DLN) and expression of costimulatory molecules CD80, CD86, and CD40 on DCs. In contrast to LT-I, LT-IIb and LT-IIc induced less edema, cellular infiltrates, and general inflammation at the site of ID injection. Thus, LT-IIb and LT-IIc are attractive comprehensive ID adjuvants with unique characteristic that enhance humoral and cellular immunity to a co-administered protein Ag.

## Introduction

Vaccination remains one of the most efficient and cost-effective methods of combating infectious diseases [1]–[3]. Not all archetypical antigens (Ag), however, are immunogenic and induce protection. Additionally, the use of protein subunit vaccines have, in general, replaced the use of whole cell preparations or attenuated strains as immunogens. While protein subunit vaccinations are well-tolerated, these agents frequently elicit only humoral responses and often fail to induce strong protection against complex diseases such as those elicited by viruses [4], which usually require induction of CD8^+^ T cell responses or other components of the cellular immune system [5]. To enhance immune reactions to poor immunogens and to provide comprehensive immune responses (i.e., humoral and cellular components) against complex infectious diseases, adjuvants are routinely required in vaccination.

In addition to the use of adjuvants, alternative routes of vaccine administration have been utilized to enhance immune responses. Traditionally, vaccines are administered by either the intramuscular (IM) or subcutaneous (SC) route. These sites, however, lack dense populations of Ag presenting cells (APC) and, therefore, require higher concentrations of Ag to raise significant immune responses. As the skin contain numerous APC, such as Langerhans cells (LC) and dermal dendritic cells (dDC) that can initiate robust immune responses [6], the intradermal (ID) route of administration offers an attractive alternative immunization site. For example, only approximately half the normal IM dose was required to induce a protective response when individuals were vaccinated with the first FDA-approved ID influenza vaccine (Fluzone Intradermal, Sanofi Pasteur). This Ag-sparing effect was evident even when the ID vaccine was used in poor responder populations (e.g., the elderly) [6].

A major concern, however, is the choice of an appropriate adjuvant for optimizing ID vaccine outcomes. Cholera toxin (CT) produced by *Vibrio cholerae* and LT (herein referred to as LT-I), LT-IIa, LT-IIb, and LT-IIc expressed by enterotoxigenic strains of *Escherichia coli* are potent adjuvants that enhance both mucosal and systemic immunity [7]–[9]. CT and LT-I are members of the type I subfamily of heat-labile enterotoxins (HLT); the type II HLT subfamily is composed of LT-IIa, LT-IIb, and LT-IIc [10]. The two HLT subfamilies are classified based on antigenic neutralization; antiserum against LT-I neutralizes the activity of CT, but not the activities of any of the type II HLT. All HLT belong to the AB_5_ toxin family and contain a single enzymatically active A polypeptide that is noncovalently linked an array of pentameric B polypeptides. The A polypeptide catalyzes the ribosylation of the G_sα_ subunit thereby constitutively activating adenylate cyclase and elevating cAMP in the intoxicated cell [11]. The pentameric B-subunits are responsible for binding various gangliosides on cell surfaces [12]. While the A subunits of the type II HLT are similar (∼75% homology) to each other, the B subunits share only ∼50% homology [12].

In mucosal immunization models, LT-IIa, LT-IIb, and LT-IIc have been shown to induce distinctive patterns of immunologic responses in comparison to those patterns induced by CT or LT-I [9], [10]. While type I HLT typically elicit Th2 responses in intranasal immunization models, the type II HLT typically induce a more balanced Th1/Th2 response [13]. Additionally, LT-IIc appear to induce a predominant Th1 response (TDC unpublished). Furthermore, LT-I, CT, and LT-IIa greatly accelerate apoptosis in mitogenically driven CD8^+^ T cells (human and mice) *in vitro* while LT-IIb and LT-IIc lacks this activity ([13], TDC unpublished). The distinctive immunomodulatory properties of the type II HLT have been attributed to their binding affinities for various non-GM1 gangliosides that are located on immune cell populations [7].

While LT-IIb and LT-IIc have been demonstrated to be potent mucosal adjuvants [9], [14], their immunostimulatory properties, when introduced by the ID route, have not been fully evaluated. Experiments reported herein revealed that LT-IIb and LT-IIc significantly enhanced levels of Ag-specific antibodies while inducing significantly less inflammation at the injection site in comparison to LT-I, a prototypical type I HLT, when employed as an ID adjuvant. Additionally, administration of LT-IIb and LT-IIc induced a more prolific CD8^+^ T cell response in comparison to LT-I and unadjuvanted mice and exhibited superior protection against a challenge with *Listeria monocytogenes*, an intracellular pathogen. These data indicate that LT-IIb and LT-IIc have great potential as clinical adjuvants for use in ID vaccines.

## Methods

### Mice and immunizations

Female 8–12 week C57Bl/6J mice (Jackson Laboratory, Bar Harbor, ME) were used. Prior to immunizing, mice were anesthetized using ketamine (75 mg/kg) and xylazine (10 mg/kg). Hair on the lower flank was removed with electric clippers and the skin sterilized with 70% isopropanol. Injections were performed using a 29G needle and 0.5 cc insulin syringe (BD, Franklin Lakes, NJ) with the bevel side up. Chicken egg ovalbumin (OVA) (Sigma, St. Louis, MO), a model Ag, was used at 50 µg in the presence or absence of HLT adjuvants diluted to a final volume of 20–30 µl using phosphate buffered saline (PBS). Mice were euthanized by CO_2_ asphyxiation and/or cervical dislocation at the termination of the experiment.

### Ethics statement and veterinary care

All experiments employing animals at The University at Buffalo (Approval #MIC01010Y) and at The University of Sao Paulo (Approval #053/10) were approved by each university’s Institutional Animal Care and Use Committee (IACUC). Animal facilities at both institutions are administered by full-time professional veterinarians, who are available for advice and assistance. The animal facility at The University at Buffalo is accredited by the American Association for the Accreditation of Laboratory Animal Care (AALAC), which complies with NIH policy, the Animal Welfare Act, and all other applicable federal, state, and local laws.

### Antigen and adjuvant preparations

Recombinant LT-IIb and LT-IIc were purified using nickel affinity and gel chromatography [9]. LT-I was prepared using a modification of a previously described method [9]. Cholera toxin-negative *V. cholerae* JBK70, which was transformed with plasmid pML19 [15] expressing LT-I, was cultured for 18 hr in an incubator/shaker (37°C, 300 rpm) in LB broth containing 200 µg/ml ampicillin (RPI, Prospect, IL). Culture supernatant, obtained by centrifugation (17,000×g, 20 min, 4°C), was sterilized by filtration using a 0.22 µm vacuum filter (Corning, Tewksbury, MA). Sodium azide (Sigma, St. Louis, MO) was added at 0.01% and phenylmethanesulfonyl fluoride (Sigma) at 0.25 mM final concentration and proteins in the media were precipitated overnight at 4°C with 400 mg/ml ammonium sulfate (Ameresco, Solon, OH). Proteins were recovered from the media by centrifugation at 17,000 g for 45 minutes at 4°C, resuspended in and dialyzed against PBS containing PMSF and sodium azide. Soluble proteins were passed over Immobilized D-Galactose resin (Pierce, Rockford, IL) and LT-I was eluted from the column with 0.3 M D-Galactose (Fisher Sci, Pittsburg, PA). Additional purification of LT-I was performed using HiPrep 26/60 Sephacryl S-100 (GE Healthcare, Uppsala, Sweden). Protein concentrations were determined using a Micro BCA Protein Assay Kit (Pierce, Rockford, IL). If needed, contaminating LPS was removed from all proteins using Detoxi-Gel (Pierce). LPS contamination was ≤0.03 ng/µg protein in all preparation of enterotoxin, as determined by use of a Limulus Amoebocyte Lysate Endochrome kit (Charles River Endosafe, Charleston, SC).

### Skin edema analysis

The extent of skin edema was determined by taking two orthogonal measurements (M_1_ and M_2_) of the induration diameter. Edema volume was calculated as M_1_×M_2_×M_2_, where M_1_≥M_2_.

### Histology

Skin sections containing the injection site were surgically removed from mice that had been euthanized 48 hr after immunization. Sections were fixed in 10% formalin and paraffin-sectioned for H&E staining. The numbers of infiltrating cells at 600x magnification in 5 fields/skin sections were counted by a veterinary pathologist.

### Myeloperoxidase assay

The myeloperoxidase (MPO) activity of ID injected skin sections was determined spectrometrically [16]. Briefly, 100 mg of minced skin containing the injection site were homogenized (2×45 sec, 0°C) in 1.9 ml of sodium phosphate buffer (0.02 M NaH_2_PO_4_, 0.015 M Na_2_EDTA, 0.1 M NaCl, pH 4.7) using a disperser device (IKA, Wilmington, NC) and centrifuged (10,000×g, 10 min, 4°C). The pellets were resuspended in 1.5 ml of 0.05 M sodium phosphate buffer (pH 5.4) that contained 0.5% hexadecyltrimethylammonium bromide, freeze-thawed (3x), and the suspension were re-centrifuged. Duplicates of each sample (50 µl/well) were mixed with 150 µl of BD OptEIA TMB substrate reagent and incubated (37°C, 5 min). The reactions were terminated by addition of 150 µl/well of 4M H_2_SO_4_. Absorbance of the reaction was determined at 450 nm. Results of the MPO assays were expressed as OD_450 nm_/mg of total protein.

### Ab ELISA

Levels of OVA-specific serum Ab were measured using ELISA [8]. Anti-mouse IgG capture Ab and enzyme-conjugated detection Ab were purchased from SouthernBiotech (Birmingham, AL). Murine IgG standard reference serum was purchased from MP Biomedicals (Solon, OH).

### Flow cytometry and Ab

Cells were suspended in FACS buffer (PBS, 0.1% heat-inactivated fetal calf serum, 0.01% sodium azide), stained for extracellular markers, and fixed in 4% paraformaldehyde. Ab were obtained from eBioscience (San Diego, CA), Biolegend (San Diego, CA), or BD Biosciences (San Jose, CA): TCRβ (Clone H57-597), CD8α (Clone 53-6.7), CD11c (Clone N418), MHC-II (Clone M5/T14.15.2), CD80 (Clone 16-10A1), CD86 (Clone GL1), CD40 (Clone 1C10). OVA-specific dextramer was purchased from Immudex (Copenhagen, Denmark). Aqua LD stain (Life Technologies, Grand Island, NY) was employed for live dead differentiation. Data was captured using FACSCalibur (BD Biosciences), LSR-II (BD Biosciences), or LSR-Fortessa (BD Biosciences) and analyzed using FlowJo (Treestar, Ashland, OR).

### *Listeria monocytogenes* challenge

Recombinant *L. monocytogenes* (rLM-OVA) expressing OVA_134–387_ (strain DMX 09-082) was purchased from DMX Inc. (Philadelphia, PA). rLM-OVA were cultured in Brain heart infusion (BHI) broth containing 100 µg/mL erythromycin. Cultured bacteria were washed with PBS and the washed bacteria frozen at −80°C in PBS at a known cfu/ml. Immunized mice were injected with one LD_50_ (5×10^6^ CFU) i.v. [17] and euthanized after 3 days to enumerate CFU in the spleen. Serial dilutions of spleen homogenates were inoculated onto BHI agar plates containing 100 µg/mL erythromycin, which were incubated at 37°C for two days prior to colony counting.

### Dendritic cell analysis

Inguinal lymph nodes that had been isolated 24 hr after ID immunization were homogenized through a 40 µM nylon mesh (Corning). Cells were washed in FACS buffer, stained, and analyzed by flow cytometry for surface marker expression, as described above.

### Statistical analysis

All data were analyzed using Graphpad Prism (GraphPad Software, Inc., La Jolla, CA).

## Results

### Differential inflammatory responses to ID-administered LT-IIb, LT-IIc, and LT-I

**Figure 1:**
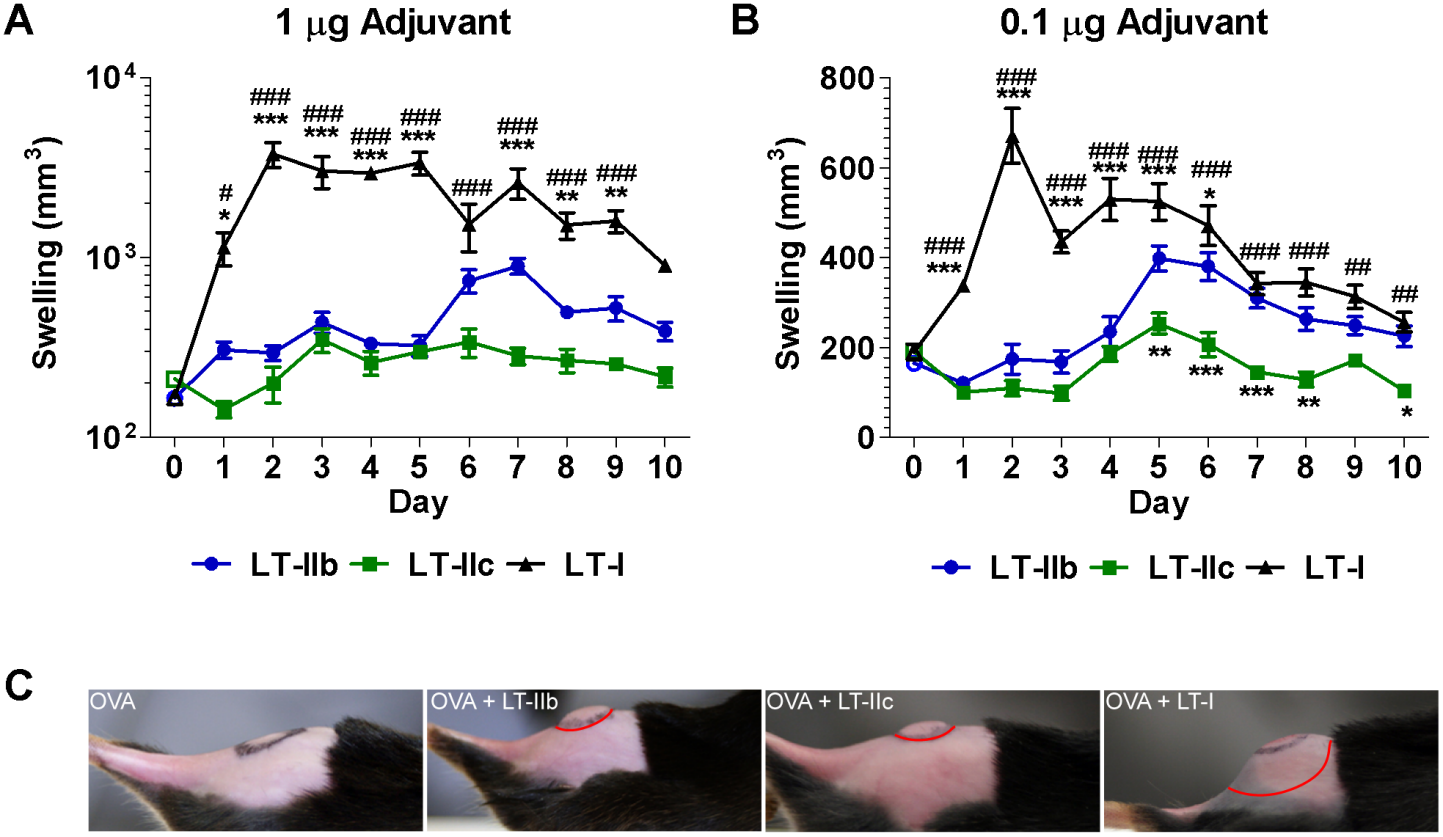
LT-IIb and LT-IIc induce less swelling than LT-I at the site of injection. Kinetics of swelling volume measured to day 10 after primary immunization with 50 µg of OVA and (A) 1.0 µg or (B) 0.1 µg of HLT adjuvants. Swelling at day 0 represents the size of the induration immediately after injection (open symbols). (C) Side profile of injection-associated swelling 24 hr after ID administration of 50 µg OVA +/−0.5 µg HLT adjuvant. Injection induration margins (black) and swelling margins (red). Data shown (n = 8) as a representative of two independent experiments. *Statistical Analysis:* (A, B) Two-way ANOVA with Bonferroni post-test compared to LT-IIb (*) or LT-IIc (#). *P≤0.05; **P≤0.01; ***P≤0.001 or respective symbol. Results shown as the arithmetic mean with error bars denoting SEM.

Patient compliance has a critical impact on the actual success of an adjuvanted vaccine. Thus, a desirable adjuvant should induce only minimal amounts of inflammation. While ID administration of vaccines has numerous benefits including Ag sparing, this immunization route is highly susceptible to local adverse reactions. Therefore, it is critical to determine the occurrence of local adverse reactions caused by novel adjuvants prior to further investigations and clinical consideration. To evaluate the inflammatory properties of the three HLT, injection-site inflammatory reactions were analyzed grossly and histologically after a single dose of OVA co-administered by the ID route with LT-IIb, LT-IIc, or LT-I. At the 1.0 µg dose of the HLT adjuvants, LT-IIb and LT-IIc induced 10-fold less swelling over the injection site during the first 5 days, in comparison to the inflammatory responses observed in mice that received LT-I (**Fig.** **1A**). LT-IIc induced a slightly lesser reaction than LT-IIb and this difference was accentuated at the 0.1 µg dose (**Fig.** **1B**). Additionally, a conspicuous and reproducible biphasic inflammatory reaction that separated the primary induration formation at day 2 from a secondary peak at ∼day 5 with both 1.0 µg and 0.1 µg doses of HLT adjuvants was observed (**Fig. 1A, B**). At the 0.1 µg dose, LT-IIc elicited only a slight inflammatory response at the time point in which the stronger secondary responses (day 5–10) for LT-IIb and LT-I were noted (**Fig.** **1B**). Furthermore, the greatest difference in the swelling responses of LT-I and of the two type II HLT adjuvants occurred during the early (day 1–3) primary reaction (**Fig. 1A, B**). After 24 hr, LT-IIb and LT-IIc induced a level of swelling that was essentially the size of the injection induration, while the swelling induced by LT-I greatly exceeded the injection induration margins (**Fig.** **1C**).

**Figure 2:**
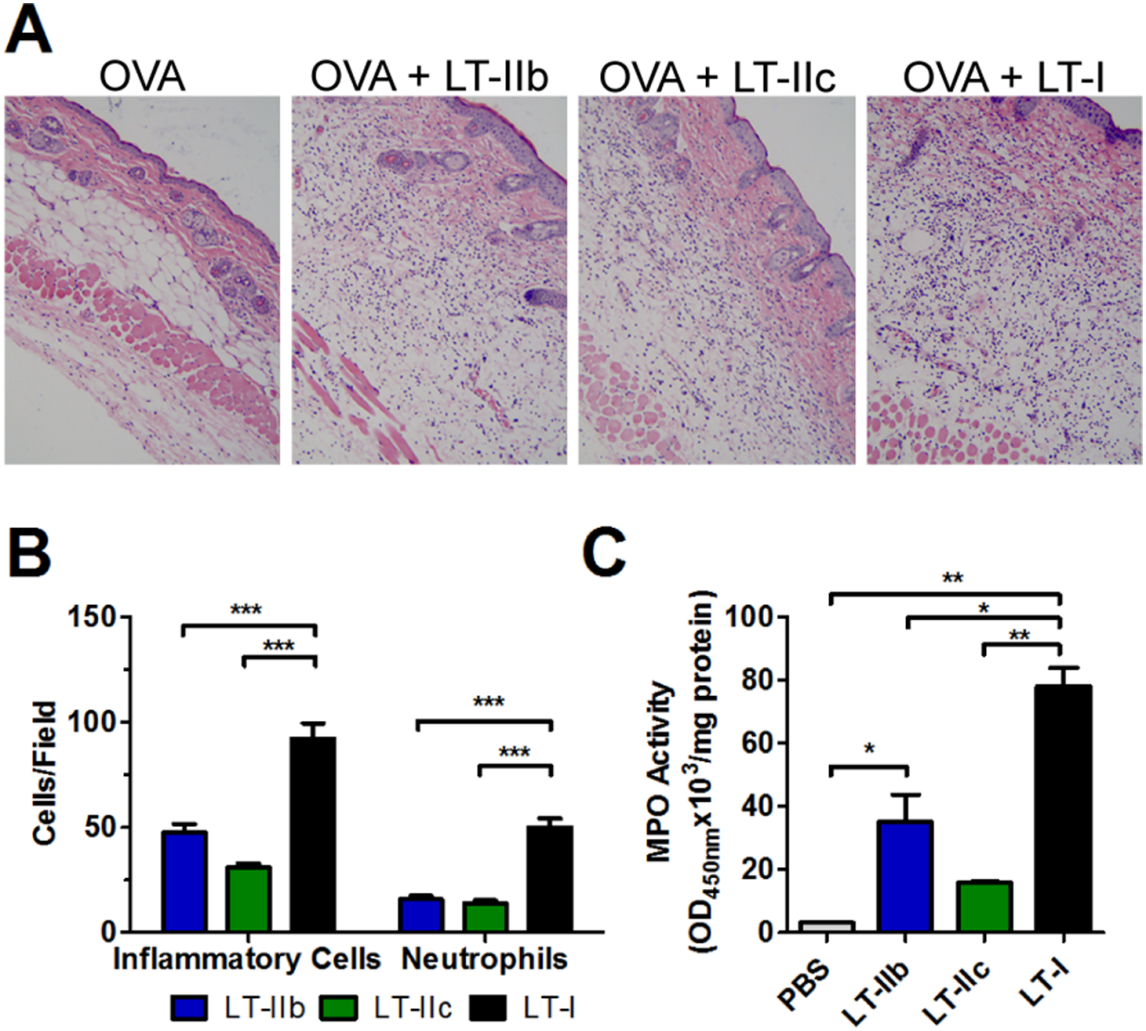
LT-IIb and LT-IIc induce less inflammatory neutrophil infiltrate than LT-I at the site of injection. (A) H&E stained sections of injection site cutaneous tissue after 48 hr with 0.5 µg of HLT adjuvant (100x magnification). (B) Visual quantification of infiltrating cells and neutrophils per high field (600x, 5 fields, n = 5) at 48 hr post-injection with 1 µg of HLT adjuvant. (C) Myeloperoxidase reaction in skin homogenates obtained after 48 hr post-injection with 1.0 µg of HLT adjuvant. Data shown (n = 2) as a representative of two independent experiments. Statistical Analysis: (B, C) One-way ANOVA with Bonferroni post-test. *P≤0.05; **P≤0.01; ***P≤0.001.

Histologically, ID administration of OVA in the presence of LT-IIb, LT-IIc, or LT-I increased immune cell infiltrates into the injection site at 48 hr post-vaccination, in comparison to unadjuvanted OVA (**Fig.** **2A**). Mice receiving LT-I, however, had statistically more inflammatory cell infiltrates of which the majority were neutrophils (**Fig.** **2B**). Moreover, the least numbers of infiltrating neutrophils were detected in mice administered with LT-IIc (**Fig.** **2C**). To confirm the histological quantification of infiltrating neutrophils, the levels of myeloperoxidase (MPO) was determined in homogenates of skin at the injection sites. In comparison to the PBS control, LT-I and LT-IIb induced statistically greater amounts of MPO enzymatic activity at the ID injection site tissue at 48 hr post-immunization. Furthermore, more MPO activity was observed in mice that had received LT-I in comparison to the activity measured in mice that had received either LT-IIb or LT-IIc (**Fig.** **2C**). Taken together, the results confirmed that LT-IIb and LT-IIc induced significantly less inflammation at the ID injection site than did LT-I, an effect that has desirable clinical implications.

### Enhancement of humoral responses by ID-administered LT-IIb, LT-IIc, and LT-I

**Figure 3:**
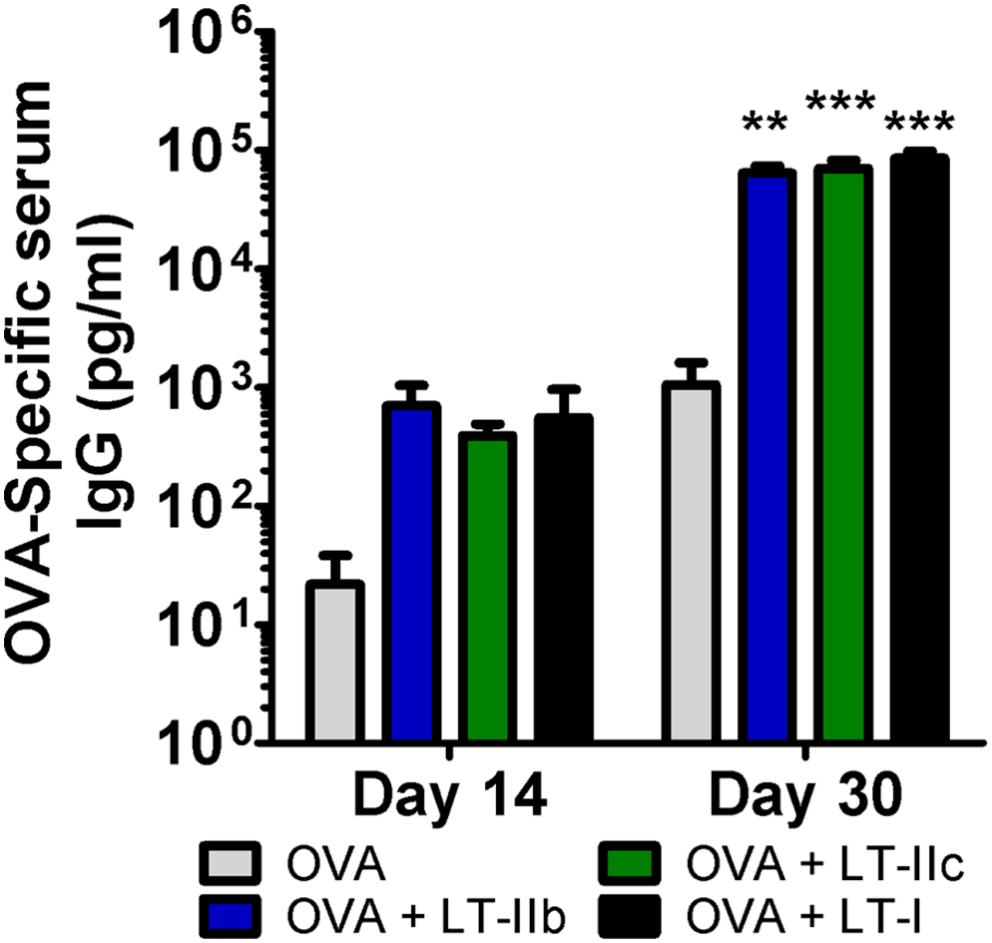
LT-IIb and LT-IIc enhance humoral responses. Total OVA-specific serum IgG levels on day 14 and 30 post-vaccination. Mice were immunized with 50 µg OVA +/−1.0 µg HLT. Data shown (n = 8) as a representative of two independent experiments. Unpooled serum was analyzed in duplicate by ELISA. Statistical analysis: One-way ANOVA with Bonferroni post-test compared to OVA. *P≤0.05; **P≤0.01; ***P≤0.001. Results shown as the arithmetic mean with error bars denoting SEM.

Generation of a robust Ag-specific Ab response is a core characteristic of an effective adjuvant. Serum IgG responses to the model Ag OVA were analyzed after ID vaccination using a 2-dose regimen at 14 day intervals. After two doses, mice receiving LT-IIb, LT-IIc, and LT-I adjuvants exhibited similar enhancements to OVA-specific serum IgG that were two orders of magnitude greater than levels induced by immunization with OVA in the absence of an adjuvant (**Fig.** **3**). Remarkably, and despite their reduced inflammatory profiles, LT-IIb and LT-IIc, when administered by the ID route, enhanced the levels of Ag-specific Ab that were equivalent to the levels induced by the more inflammatory LT-I.

### Differential enhancements of CD8^+^ T cell responses by LT-IIb, LT-IIc, and LT-I

**Figure 4:**
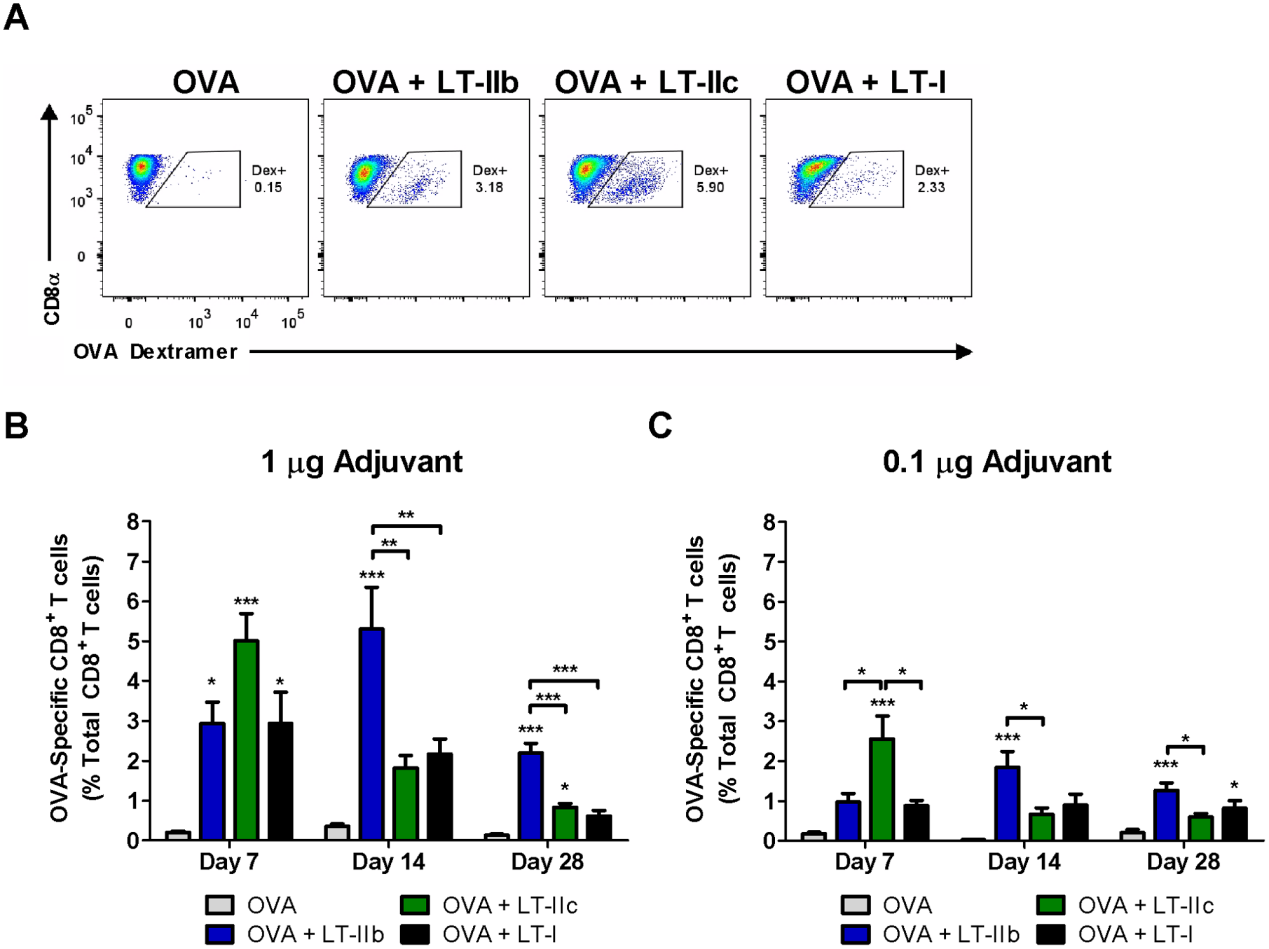
LT-IIb and LT-IIc enhance antigen-specific CD8^+^ T cell expansion after immunization, but with different kinetics. (A) Representative flow analysis of OVA-specific CD8^+^ T cells identified by MHC-dextramers 7 days after 1.0 µg HLT adjuvanted immunization. Cells gated on live TCRβ^+^CD8^+^ cells. (B) OVA-specific CD8^+^ T cells from PBMCs after 7, 14, and 28 days post-immunization utilizing 1.0 µg and (C) 0.1 µg of HLT adjuvants. Data shown (n = 8) as a representative of two independent experiments. Statistical analysis: One-way ANOVA with Bonferroni post-test compared to OVA, unless otherwise noted. *P≤0.05; **P≤0.01; ***P≤0.001. Results shown as the arithmetic mean with error bars denoting SEM.

CD8^+^ T cells are a crucial component of a cellular immune response and are essential for the clearance of many intracellular pathogens. To examine the quantitative enhancements to the CD8^+^ T cell response induced by the HLT adjuvants, an OVA-specific dextramer was utilized to enumerate Ag-specific CD8^+^ T cells after a single immunization of OVA in the presence or absence of the adjuvants (**Fig.** **4A**). At 1 week post-vaccination, all groups of mice that had received ID immunizations with 1.0 µg of an HLT adjuvant exhibited statistically significant enhancements in OVA-specific CD8^+^ T cell expansion in the peripheral blood mononuclear cell (PBMC) compartment when compared to mice that had been administered only OVA (**Fig.** **4B**). Additionally, by day 7, LT-IIc induced a greater CD8^+^ T cell response when compared to the other HLT. This difference was accentuated when 0.1 µg of the HLT adjuvants was employed (**Fig.** **4C**). Surprisingly, the Ag-specific CD8^+^ T cell response elicited by LT-IIb was strikingly different from the CD8^+^ T cell responses elicited by either LT-IIc or LT-I. Whereas mice that were adjuvanted with either LT-IIc or LT-I exhibited a clear contraction of CD8^+^ T cell numbers from day 7 to day 14 post-immunization, the numbers of these cells was increased in mice adjuvanted with LT-IIb during this same period at both the 1.0 µg and at 0.1 µg doses (**Fig.** **4** **, B and C**). Additionally, the CD8^+^ T cell response induced by LT-IIb on day 14 was equivalent to the early response induced by LT-IIc on day 7. Furthermore, mice adjuvanted with LT-IIb sustained the numbers of CD8^+^ T cells at levels that were two-fold higher at day 28 in comparison to the LT-IIc and LT-I adjuvant groups.

**Figure 5:**
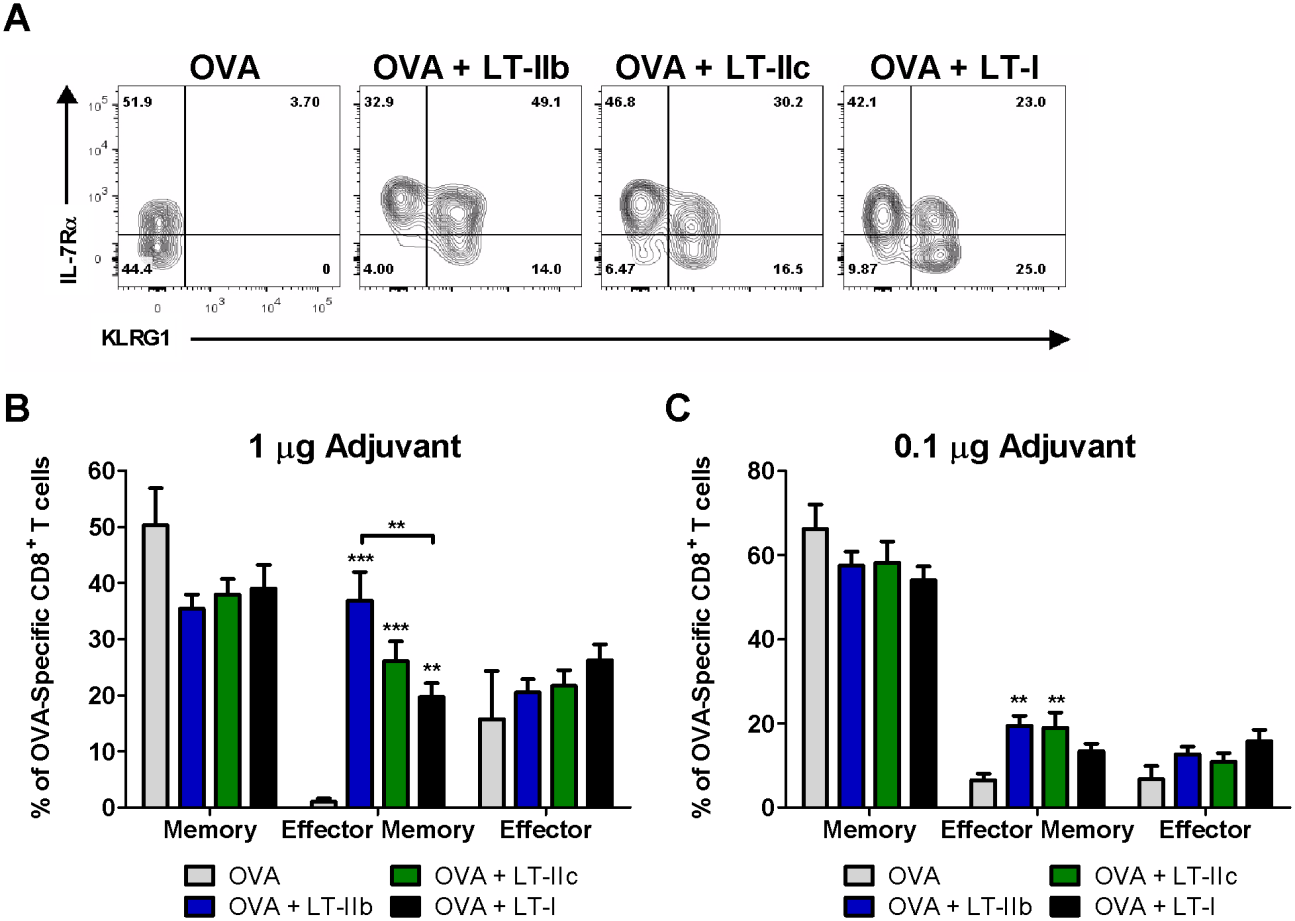
LT-IIb and LT-IIc enhance the formation of IL-7Rα^+^KLRG1^+^ effector memory CD8^+^ T cells. (A) Representative IL-7Rα and KLRG1 staining of OVA-specific CD8^+^ T cells 28 days after primary immunization with 1.0 µg HLT adjuvants. (B) Percentages of memory (IL-7Rα^+^KLRG1^−^), effector memory (IL-7Rα^+^KLRG1^+^), and effector (IL-7Rα^−^KLRG1^+^) OVA-specific CD8^+^ T cells 28 days post-immunization utilizing 1.0 µg and (C) 0.1 µg of HLT adjuvants. Data shown (n = 8) as a representative of two independent experiments. Statistical analysis: One-way ANOVA with Bonferroni post-test compared to OVA alone. *P≤0.05; **P≤0.01; ***P≤0.001. Results shown as the arithmetic mean with error bars denoting SEM.

Since CD8^+^ T cell populations typically contract soon after a primary response [18], the elevated numbers of OVA-specific CD8^+^ T cells that were sustained at day 28 in the group of mice that had been adjuvanted with LT-IIb was unanticipated. Thus, a further analysis of the phenotype of the CD8^+^ T cells was performed to identify the constituents of these surviving cells and to evaluate the quality of the memory response. Utilizing IL-7Rα (CD127), a marker of memory, and KLRG1, a marker of terminal differentiation, quantification of long lived memory (IL-7Rα^+^KLRG1^−^) and effector memory (IL-7Rα^+^KLRG1^+^) cells and short lived effector cells (IL-7Rα^−^KLRG1^+^) in the OVA-specific CD8^+^ T cell population was performed (**Fig.** **5A**). While no significant differences were observed in the percentages IL-7Rα^+^KLRG1^−^ memory cells or IL-7Rα^−^KLRG1^+^ effector cells, dramatic differences were identified at day 28 post-immunization in the IL-7Rα^+^KLRG1^+^ effector memory population. In comparison to mice that had received only OVA, enhanced percentages of effector memory cells were detected in mice that had been administered 1.0 µg doses of either LT-I, LT-IIb, or LT-IIc (**Fig.** **5B**). Only the groups of mice that had been ID immunized with OVA in the presence of LT-IIb or LT-IIc, however, had statistically significant increases in the effector memory population at the 0.1 µg dose (**Fig.** **5C**) in comparison to mice that had received OVA in the absence of an adjuvant. Importantly, the numbers of effector memory cells was increased by 10% and 20% in mice that had received 1.0 µg of LT-IIb in the surviving OVA-specific CD8^+^ T cell population than did mice that had received LT-IIc or LT-I, respectively (**Fig.** **5B**). The enhanced percentages of highly functional and long lived effector memory cells in conjunction with superior numbers of circulating Ag-specific CD8^+^ T cells (**Fig.** **4** **, B and C**) suggested that of the three HLT, LT-IIb had the greatest capacity to induce the formation of a robust cellular immune memory response.

### Differential Ag-specific clearance of *Listeria monocytogene*s by LT-IIb, LT-IIc, and LT-I

**Figure 6:**
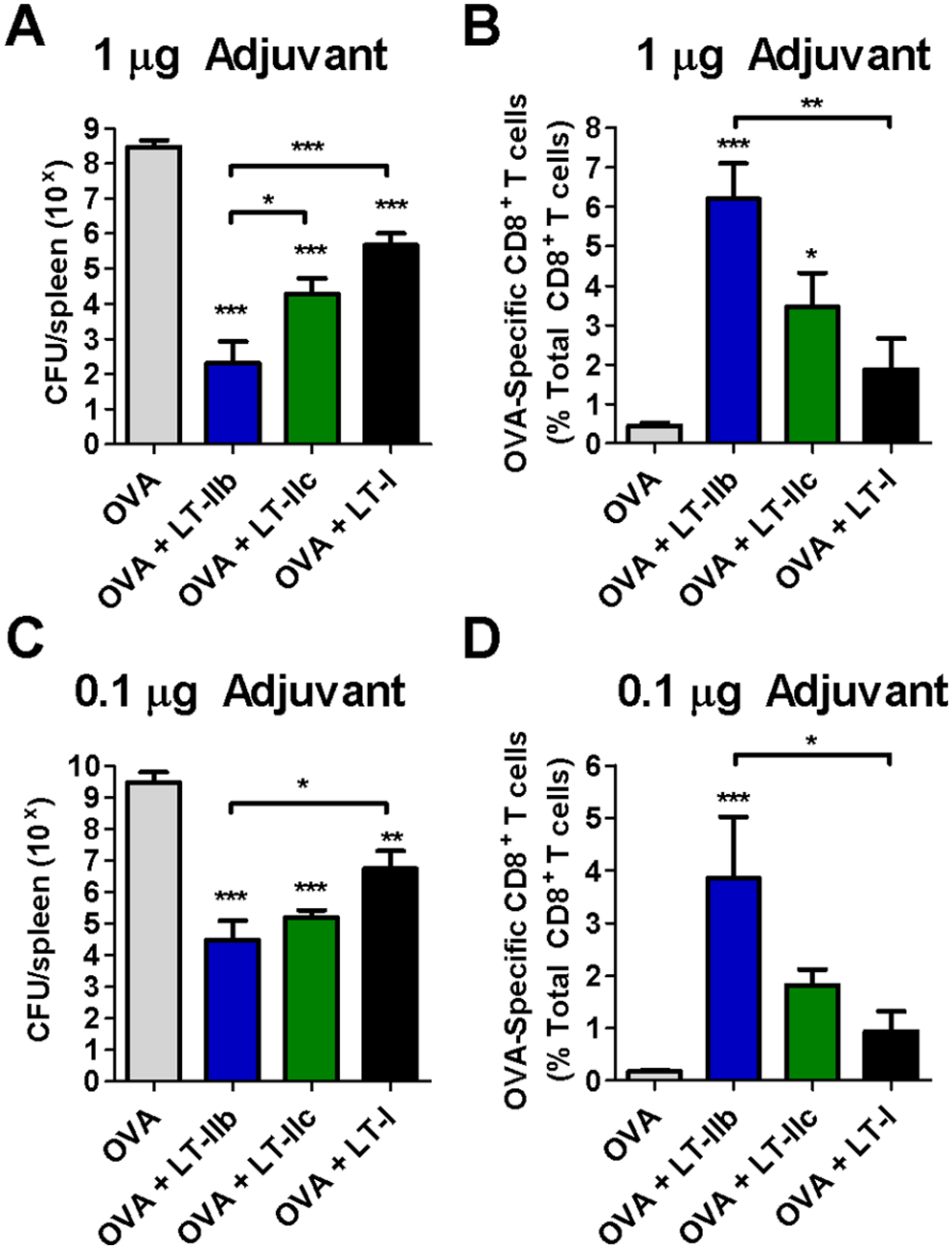
LT-IIb exhibits superior clearance of rLM-OVA and CD8^+^ T cell recall. Mice immunized with OVA +/− HLT adjuvants were challenged with 5×10^6^ CFU of recombinant OVA-expressing *Listeria monocytogenes* (rLM-OVA) 30 days post-vaccination. (A) CFU enumerated from homogenized spleens 3 days post rLM-OVA challenge of mice immunized with OVA and adjuvanted with 1.0 µg of HLT and (B) OVA-specific CD8^+^ T cell secondary recall 3 days post challenge. Cells gated on live OVA dextramer^+^TCRβ^+^CD8^+^ cells. (C) Splenic CFU and (D) OVA-specific CD8^+^ T cell recall as before in mice immunized OVA and 0.1 µg of HLT adjuvant. Data shown (n = 8) as a representative of two independent experiments. Statistical analysis: One-way ANOVA with Bonferroni post-test compared to OVA alone unless otherwise noted. *P≤0.05; **P≤0.01; ***P≤0.001. Results shown as the geometric (A, C) or arithmetic (B, D) mean with error bars denoting SEM.

To evaluate the qualitative and functional enhancements to the CD8^+^ T cell population, a well-established OVA-expressing *L. monocytogenes* (rLM-OVA) challenge model was employed. Thirty days after a single ID dose of OVA in combination with LT-I, LT-IIb, or LT-IIc, mice were challenged with rLM-OVA (5×10^6^ CFU). After 3 days, the challenged mice were euthanized to enumerate CFU of the intracellular pathogen in the spleen. All mice that had received OVA adjuvanted with either LT-I, LT-IIb, or LT-IIc had lower splenic CFU of the bacterium when compared to mice receiving OVA in the absence of an adjuvant. The strongest protection, however, was attained in mice that had received LT-IIb as an adjuvant at either 0.1 µg or 1.0 µg doses (**Fig. 6** **, A and C**). A ∼7-log reduction in the CFU of *rLM-OVA* was observed in the spleens of mice that had received OVA and 1.0 µg of LT-IIb in comparison to mice that had received only OVA (**Fig.** **6A**). Additionally, when used as an ID adjuvant, the spleens of LT-IIb-administered mice had a 2-log and 3-log lower CFU of *rLM-OVA* in comparison to the CFU from the spleens of mice that had been ID immunized with OVA in combination with either LT-IIc or LT-I, respectively. Accordingly, LT-IIb adjuvanted mice exhibited a superior secondary expansion of OVA-specific CD8^+^ T cells after challenge with rLM-OVA in comparison to the OVA-specific CD8^+^ T cell population in mice that had been co-administered OVA in combination with either LT-I or LT-IIc or in mice that had received only OVA (**Fig.** **6B**). Similar trends were observed in clearance responses when the dose of the three HLT adjuvants was reduced to 0.1 µg. At this lower dose, however, LT-IIb did not proffer a statistically better clearance of the intracellular pathogen than did LT-IIc (**Fig.** **6C**). The secondary recall response of OVA-specific CD8^+^ T cells for LT-IIb at the 0.1 µg dose, however, was superior to all other experimental groups (**Fig. 6D**).

In summary, while LT-IIb, LT-IIc, and LT-I enhanced the Ag-specific cellular immune response and clearance of an intracellular pathogen (rLM-OVA) when used as ID adjuvants, LT-IIb induced the strongest resistance against challenge at a point 30 days after a single immunization and exhibited a superior secondary Ag-specific CD8^+^ T cell recall at doses of 1.0 µg and at 0.1 µg.

### Modulation of DC in the draining lymph node by LT-IIb, LT-IIc, and LT-I

**Figure 7:**
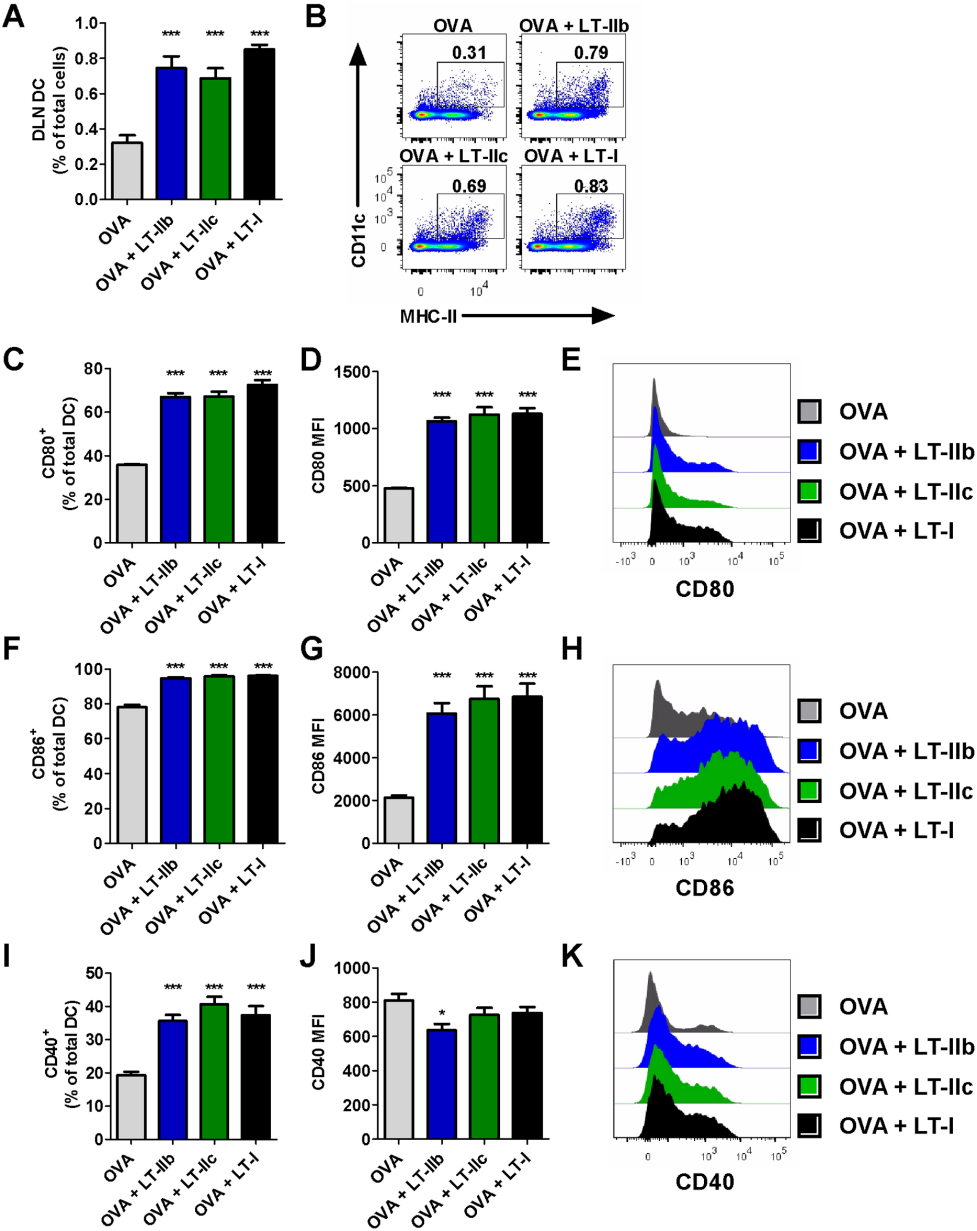
Enhancement of dendritic cell migration and expression of costimulatory molecules in the cutaneous draining inguinal lymph node by LT-IIb, LT-IIc, and LT-I after ID vaccination. (A) Percentages of live CD11c^+^MHC-II^+^ dendritic cells in the total cell population of the inguinal lymph node, and (B) representative gating of cells 24 hr post-immunization with 50 µg of OVA and 1.0 µg of HLT adjuvants. (C) Percentages of positive staining DC, MFI, and representative histograms of (C–E) CD80, (F–H) CD86, and (I–K) CD40. Data shown (n = 8) with MFI denoting geometric means of fluorescence where applicable. Statistical Analysis: (A–K) One-way ANOVA with Bonferroni post-test compared to OVA alone unless otherwise noted. *P≤0.05; **P≤0.01; ***P≤0.001. Error bars denote SEM.

To evaluate the role of APC for the adjuvant effects of LT-IIb and LT-IIc on humoral and cellular immune responses, DC in the cutaneous DLN (inguinal node) were examined after ID administration of OVA in the presence or absence of the HLT adjuvants. Twenty-four hours after immunization with OVA and 1.0 µg of LT-I, LT-IIb, or LT-IIc, DC numbers in the DLN increased by approximately two-fold in the adjuvanted mice in comparison to the numbers of DC in mice immunized solely with OVA (**Fig.** **7** **, A–B**). Additionally, DC in the DLN of adjuvanted mice all exhibited an enhanced expression of costimulatory molecules CD80 (**Fig. 7** **, C–E**) and CD86 (**Fig.** **7** **, F–H**). Furthermore, the percentage of CD40^+^ DC in the DLN was increased significantly in mice adjuvanted with either LT-I, LT-IIb, or LT-IIc when compared to the percentage of CD40^+^ DC in mice that had received OVA in the absence of an adjuvant (**Fig. 7**, **I–K**).

## Discussion

Clinically acceptable adjuvants must have the capacity to enhance Ag-specific immune responses while inducing minimal side effects, such as irritation and inflammation. Administration of type I HLT adjuvants (e.g., CT and LT-I) by cutaneous routes, in most cases, induced significant amounts of local inflammation [19], [20]. Of the type II HLT family, only LT-IIa and LT-IIb has been evaluated as ID adjuvants. In those studies, both LT-IIa and LT-IIb were less inflammatory than LT-I [8], [21]. Additionally, only LT-IIa had been previously evaluated for the capacity to enhance CD8^+^ T cells. Those studies, however, did not include experiments to determine if the enhancements in CD8^+^ T cells had a concomitant enhancement in clearance after a pathogenic challenge. In the present work, LT-IIb and LT-IIc, when used as ID adjuvants, similarly displayed less irritation at the injection site than LT-I when evaluated by the extent of edema formation and by measurements of inflammatory infiltrates (**Fig.** **1** **,** **2**). Loss of a local inflammatory response by LT-IIb and LT-IIc, however, was not reflected in a reduction in adjuvant properties. To the contrary, LT-IIb and LT-IIc enhanced Ag-specific immune responses to levels that were equivalent or exceeding those of LT-I.

Studies describing the inflammatory behavior of MF59 and alum by IM routes [22], [23] and of saponin, incomplete Freund’s adjuvant, and monophosphoryl lipid A by ID routes [24], have indicated that neutrophils are the major infiltrating cell population at the site of injection in the few hours after administration. Despite the rapid response and ability of neutrophils to sequester Ag and migrate to the DLN, Calabro et al. [22] and Lu and Hogenesch [23] showed that immunodepletion of neutrophils had no impact upon the adjuvant activity of MF59 or alum, respectively. In agreement, LT-IIc, in comparison to LT-IIb and LT-I, induced the lowest neutrophilic infiltrate and generated a robust humoral response, which were equivalent to those engendered by LT-IIb and LT-I. This observation suggests that the adjuvant activity of HLT is dissociable from their inflammatory activities. Indeed, some modifications of wild-type HLT to reduce unwanted inflammation have not drastically reduced their capacity to enhance the Ag-specific humoral responses [8], [19], [25], [26].

Analysis of the cells in the draining lymph nodes (dLN) revealed that all three HLT adjuvants enhanced DC costimulatory expression of CD80, CD86, and CD40 (**Fig. 7**). Additionally, the number of DC in the DLN doubled in comparison to those numbers of DC in mice that received unadjuvanted immunizations. These increased DC numbers are most likely due to enhanced migration of DC residing in the skin, although their exact phenotype requires additional investigation. Activation and migration of DC could be the general mechanism by which these HLT adjuvants modulate immune responses. Indeed, Zoeteweiji et. al. [19] demonstrated that LT-I induces the migration of cutaneous DC when delivered ID, and that APC migration was independent of GM1 binding. The studies presented herein corroborated the model that GM1 binding is, indeed, dispensable for HLT-induced APC migration and activation, since LT-IIb has no known affinity for GM1 and LT-IIc has only moderate affinity for this ubiquitous ganglioside [27]. Yet, the precise mechanisms by which these HLT promote the unique patterns of CD8^+^ T cell responses have not been elucidated. Therefore, further investigation into the priming environment induced by these adjuvants is warranted.

Over the past several decades, the development of targeted and specific types of immune responses (e.g., Th1, Th2, or Th17) elicited by vaccines have been shown to be critical for optimal and effective clinical results [28]. Therefore, a determination of the specific profiles and types of the immune response elicited by a particular adjuvant is critical for their optimal deployment. In this regard, the difference in the kinetics of the CD8^+^ T cell response by LT-IIb and LT-IIc is particularly intriguing. While LT-IIc induces a more rapid primary Ag-specific CD8^+^ T cell expansion, the slower kinetics of LT-IIb likely enables a larger number of these cells to survive during the contraction phase and to form highly functional long-lived memory cells (**Fig.** **4** **,** **5**). Indeed, rapid terminal differentiation and early expression of effector functions in a CD8^+^ T cell negatively correlates with survival [18]. The memory CD8^+^ T cells that are induced by LT-IIb appear to be extremely responsive and could rapidly expand to exert functional clearance of an intracellular pathogen such as *L. monocytogenes* (**Fig. 6**). As an adjuvant for preventative vaccinations, it is clear that LT-IIb provides better memory formation and recall than does LT-IIc. In contrast, a robust and rapid primary response is beneficial in other clinical applications including therapeutic vaccination in the context of established malignancies. For the eradication of existing tumor cells, the elicitation by an adjuvanted vaccine of a robust and rapid primary response is extremely advantageous. Thus, within the type II HLT family, LT-IIb and LT-IIc have the properties of distinctive CD8^+^ T cell adjuvants, each having relevant therapeutic potential.

In summary, the entire HLT family of adjuvants demonstrates the ability to induce robust immune responses to otherwise poorly immunogenic antigens. However, studies herein and published previously clearly demonstrate that these immune responses are different between the type I and type II HLT families. While type I HLTs (LT-I and CT) induce very similar responses, each type II HLT exhibits a distinct immunomodulatory profile. Therefore, the choice of a type II HLT adjuvant would allow for a more tailored immune response for a specific clinical application. Finally, to construct an even more clinically acceptable adjuvant, studies are currently underway to determine if point mutations that alter binding of the type II adjuvants are able to maintain these unique adjuvant properties while significantly reducing unwanted inflammation when employed in intradermal immunizations.

## Conclusion

The unique characteristics of the ID route, which includes dose sparing, have stimulated recent investigations for vaccine development. Discovery of new ID adjuvants that have the capacity to engage humoral and cellular immunity is a highly desirable event for vaccine design. Herein, the capacity of LT-IIb and LT-IIc, when used as ID adjuvants, to enhance both humoral and cellular immune responses without eliciting excessive local inflammation emphasizes their potential clinical importance.
